# Remifentanil attenuates sepsis-induced intestinal injury by inducing autophagy

**DOI:** 10.1080/21655979.2021.1997562

**Published:** 2021-12-11

**Authors:** Mingli Wang, Shiqi Guo, Yu Zhang, Yao Zhang, Hong Zhang

**Affiliations:** a Department of Nursing, Shengjing Hospital of China Medical University, Shenyang, Liaoning China; b Department of General Surgery, Shengjing Hospital of China Medical University, Shenyang, Liaoning Province, China

**Keywords:** Remifentanil, sepsis-induced intestinal injury, autophagy

## Abstract

Remifentanil (RFT), extensively used for general anesthesia, is a synthetic ultra-short-acting opioid used as an anti-inflammatory oxidant to alleviate a plethora of diseases. This study was designed to determine whether RFT would provide protective effects on sepsis-induced intestinal injury.The determination of cell viability and inflammation of LPS-treated IEC-6 cells influenced by RFT was conducted by Cell counting Kit-8 (CCK-8), RT-qPCR, and western blot, while the detection of LDH, diamine oxidase (DAO), and intestinal-type fatty acid binding proteins (I-FABP) was conducted for determining the intestinal cytotoxicity in these cells. The apoptosis of these cells was detected by TUNEL, with autophagy-related protein expression measured by western blot to confirm whether autophagy was activated. Finally, the aforementioned assays were conducted again after 3-Methyladenine (3-MA), an autophagy inhibitor, was used on these cells to investigate whether RFT exerted its effects on LPS-treated IEC-6 cells via modulation of autophagy.RFT alleviates LPS-induced IEC-6 cell inflammation, cytotoxicity and apoptosis, and autophagy-related proteins were expressed at higher levels when RFT was used on these cells. Nevertheless, further treatment of 3-MA weakened the restorative impacts of RFT on the inflammation, cytotoxicity and apoptosis of these cells.To conclude, this paper is the first to present evidence that RFT attenuates sepsis-induced intestinal injury by inducing autophagy, which will provide instructions for the future investigations into the use of RFT in treatment of intestinal injury.

## Introduction

Sepsis, a complex syndrome characterized by intensified inflammation in critically ill patients, has threatened hundreds and thousands of lives and incurred other complications [1–3]. This kind of heterogeneous disease that results in high morbidity and mortality presses us to explore novel therapeutic remedies for this disease [4]. The intestine acts as a promoter in inflammatory response and is essential in the pathophysiology of sepsis due to its reservoir of large amounts of bacteria [4–6]. Normal intestinal mucosal barrier, a barrier composed of intestinal epithelial cells, can prevent the attack of bacteria and wastes detrimental to human health from invading human bodies, but this maintenance can be disrupted as a result of sepsis induction [7]. Some researches have revealed potential methods including potential targets and drugs for the therapy of sepsis-induced intestinal injury [8,9]. However, it is still necessary for us to identify novel targets or drugs for the potential treatment of sepsis-induced intestinal injury.

Sedation and analgesia are common practices for the alleviation of pain, anxiety, and misery in patients who are critically ill in intensive care units to confirm smooth operations [10]. General anesthesia refers to a deep sedation featured by patients’ unresponsiveness to the agonizing stimulation or unconsciousness during the operations [10]. Remifentanil (RFT), extensively used for general anesthesia, is a synthetic ultra-short-acting opioid used as an anti-inflammatory oxidant to alleviate a plethora of diseases [11]. It can also play an important role in suppressing the production of ROS, DNA fragmentation, and the levels of interleukin 1 beta and reactive stellate [12]. RFT affects neutrophil adhesion, transmigration, and intercellular adhesion molecule expression to alleviate the inflammatory responses [13]. Additionally, RFT preconditioning played a cardioprotective effect and was able to incur autophagy and reduce apoptosis [14,15]. Furthermore, the beneficial role of RFT in improving degraded articular cartilage matrix and bone damage could potentially relieve the symptoms of osteoarthritis [16].

Autophagy is a degradation and recycling process through which cells digest its part of the reserves in cellular spaces for cell survival and homeostasis [17–19]. Under conditions where nutrients are of great deficiency, autophagy is initiated at the transcriptional and post-translational level, which is on a daily basis to maintain the normal physiological mechanisms [17]. Autophagy exerts its physiological functions by phenotypes of organisms (or tissues) with genetic deficiency of autophagy genes and the relevance of ATG proteins including microtubule-associated protein light chain 3 (LC3) family proteins [20,21]. LC3, which belongs to the three light chains (LC1, LC2, and LC3), had been previously thought as a crucial player in regulating aggregation and disaggregation of microtubules [22,23].

However, there is currently no study focusing on the therapeutic effects of RFT on the sepsis-induced intestinal injury. One study indicated the ameliorative effect of RFT on protecting the small intestine against ischemia/reperfusion injury via intestinal &delta; – and µ;-opioid receptors [24], we put forward the postulation that RFT may also attenuate sepsis-induced intestinal injury in autophagy-dependent manner. The present study was designed to determine the effects of RFT and reveal whether its mechanism in LPS-treated rat small intestinal epithelial cell line (IEC-6) was relates to autophagy.

### Materials and methods

## Cell culture

IEC-6 cells were obtained from American Type Culture Collection (ATCC) and cultured in an incubator containing 5% fetal bovine serum (FBS), 100 U/ml penicillin, and 0.1 mg/ml streptomycin at 37°C. LPS (Sigma-Aldrich, 10 μg/ml) treatment for 24 h was used on these cells for the in vitro model [4]. In LPS+ remifentanil group, RTF at the concentrations of 1 nM, 10 nM, and 100 nM was used to pre-treat IEC-6 for 5 min before LPS stimulation. In LPS + remifentanil + 3-MA group, IEC-6 cells were treated with LPS and remifentanil, and then treated with 3 MA for 2 h. 3 mM 3-Methyladenine (3-MA), an autophagy inhibitor [25], was used to investigate whether RFT exerted its effects on LPS-treated IEC-6 cells via modulation of autophagy.

CCK-8 assay

IEC-6 cells were seeded into each well of a 96-well dish at the density of 3 × 10^5^ cells/mL and cultured at 37°C. for 24 h. 10 &micro;l of CCK-8 solution (Beyotime Institute of Biotechnology, Haimen, China) was added into each well for 4 h at 37°C. Following that the optical densities were read at 490 nm.

Quantitative reverse transcription PCR (RT-qPCR)

Total RNA was isolated from the IEC-6 cells using Trizol (Invitrogen, CA, USA), followed by mRNA purification using the Nucleospin®® mRNA purification RNA II kit (Macherey-Nagel, Düren, Germany). Reverse transcription was conducted by iScript cDNA Synthesis Kit (Bio-Rad, Hercules, CA, USA) according to the manufacturer’s protocol. All reactions were conducted in triplicate, and RT-qPCR was carried out using SYBR Green Master Mix I (TaKaRa, Otsu, Shiga, Japan) on the ABI 7900 Fast Real-Time PCR System (ABI, Foster City, CA, USA). The primers are as following: Tumor necrosis factor-α (TNF-α). Forward: 5ʹ-TGAGCACAGAAAGCATGATC-3ʹ, Reverse: 5ʹ-CATCTGCTGGTACCACCAGTT-3ʹ; Interleukin (IL) −1β Forward: 5ʹ – – GACCTGTTCTTTGAGGCTGAC-3ʹ, Reverse: 5ʹ – TCCATCTTCTTCTTTGGGTATTGTT-3ʹ, IL-6 Forward: 5ʹ – AGTTTCTCTCCGCAAGA-3ʹ, Reverse: 5ʹ – GCCGAGTAGACCTCATAGTGA-3ʹ, monocyte chemoattractant protein-1 (MCP-1) Forward: 5ʹ-TCCACCACTATGCAGGTCTC-3ʹ, Reverse: 5ʹ-TGGACCCATTCCTTATTGGG-3ʹ, β-actin Forward: 5ʹ-GCTCGTCGTCGACAACGGCTG-3ʹ, Reverse: 5ʹ-CAAACATGATCTGGGTCATCTTTTC-3ʹ. 2^−ΔΔCT^ method was used to calculate the relative mRNA expression [26].

Western blot

Equal amounts of protein samples were extracted from cell lysates and subjected to 10% sodium dodecyl sulfate–polyacrylamide gel by electrophoresis and transferred to polyvinylidene fluoride membranes (Amersham Biosciences, NJ, USA). After being blocked with 5% skim milk at room temperature for 2 h, membranes were incubated with appropriate primary antibodies (anti-TLR4, sc-293,072, Santa Cruz Biotechnology; anti-p65, AF0879; anti-Bcl-2, ab196495; anti-cleaved caspase3, AF7022; anti-cleaved caspase9, AF5240; anti-ATG5, DF6010; anti-Beclin1, AF5128; anti-ATG14, AF7912; anti-p62, AF5384; Affinity, USA. anti-p-p65, ab76302; anti-Bax, ab32503; anti-LC3, ab192890; anti-GAPDH, ab8245, abcam, England.) overnight at 4°C. After washing by TBST for three times, the membranes were added with HRP-conjugated secondary antibody (ab7090, abcam, England) for 1 h. The blots were monitored by enhanced chemiluminescence (Millipore, MA, USA).

The detection of LDH

After culturing the IEC-6 cells in a 96-well cell culture dish for two days, the release of lactate dehydrogenase (LDH) in these cells was monitored by LDH detection kits (Sigma, St. Louis, MO, USA) based on the suggestions provided by the manufacturer. The LDH activity was determined by reading the absorbance at 490 nm.

Enzyme-linked immunosorbent assay (ELISA)

The expression of DAO and I-FABP in cell supernatant of IEC-6 cells was determined using ELISA assay kits (NeoBioscience Technology Co., Ltd.) by a microplate reader (MULTISKAN MK3, Thermo, San Jose, CA, USA) in line with the manufacturer’s protocol.

Terminal deoxynucleotidyl transferase dUTP nick end labeling (TUNEL) assay

The collected IEC-6 cells were fixed by 4% paraformaldehyde for 30 min and then undergone PBS washing. After that PBS containing 0.3% Triton X-100 was used to incubate cells at room temperature for 5 min. Subsequently, IEC-6 cells were incubated with 50 μL TUNEL solution (Beyotime, Shanghai, China) at 37°C for 1 h, after which PBS washing was conducted for twice and 0.5 mL Antifade Mounting Medium (Beyotime, Shanghai, China) was used to seal the sections. The apoptosis rate was observed under a fluorescence microscope.

### Statistical analysis

All experimental statistics were shown as the mean ± standard deviation (SD). Statistical analysis was performed using GraphPad Prism 8.0 (GraphPad Software, San Diego, Calif). The comparisons among multiple groups by one-way analysis of variance (ANOVA) method, followed by turkey’s test. Differences were considered statistically significant at P < 0.05.

Results

RFT alleviates LPS-induced IEC-6 cell inflammation, cytotoxicity, and apoptosis

**Figure 1.**
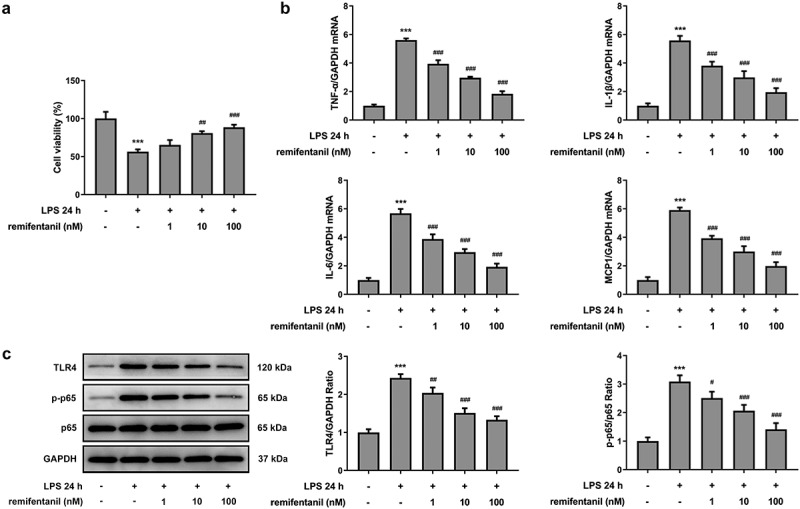
RFT alleviates the inflammation of LPS-treated IEC-6 cells. (a) The cell viability, (b) inflammatory factors and (c) inflammation-related protein expression was respectively measured by CCK-8, RT-qPCR and western blot in LPS-treated IEC-6 cells under RFT challenge. All experimental data are represented as mean ± SD. ***P < 0.001 versus control, #p < 0.05, ##p < 0.01, ### p < 0.001 versus LPS

**Figure 2.**
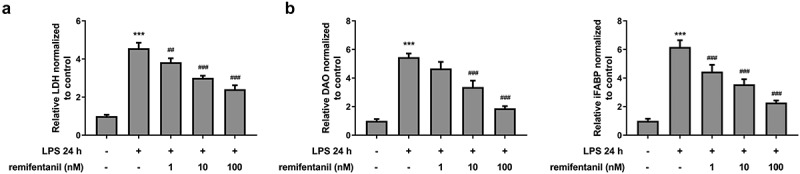
RFT reduces the intestinal cytotoxicity in LPS-treated IEC-6 cells. (a) The levels of LDH, (b) DAO and (c) I-FABP were measured to indicate the changes of intestinal cytotoxicity. The experimental data are shown as mean ± SD.***P < 0.001 versus control, ##p < 0.01, ### p < 0.001 versus LPS

**Figure 3.**
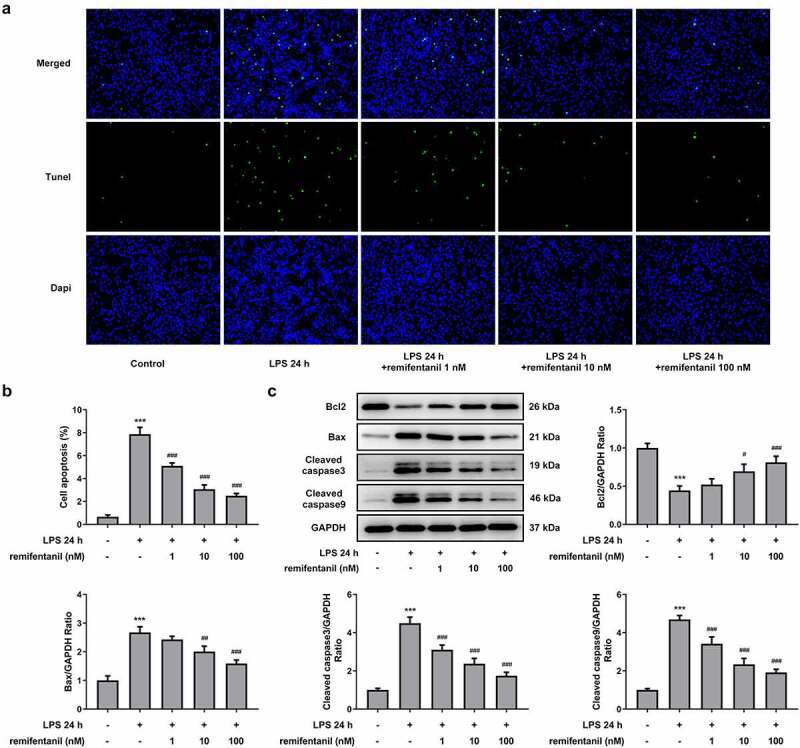
RFT alleviates the apoptosis of LPS-treated IEC-6 cells. The apoptosis of LPS-treated IEC-6 cells treated with was measured by (a) TUNEL and (b) western blot. The experimental data are shown as mean ± SD. ***P < 0.001 versus control, #p < 0.05, ##p < 0.01, ### p < 0.001 versus LPS

To examine the effects of RFT on sepsis-induced intestinal injury, we first explored its role in cell viability of IEC-6 cells. As exhibited in Figure 1a, LPS exposure dramatically damaged the cell viability of IEC-6 cells (P < 0.01), which was restored by RFT (P < 0.001). Next, regarding the inflammation that RFT may influence in sepsis-induced IEC-6 cells, RT-qPCR and western blot results which displayed decreased levels of inflammatory factors upon RFT treatment in sepsis-induced IEC-6 cells hinted the anti-inflammatory properties of RFT in LPS-induced IEC-6 cells (Figure 1b-c). ELISA detection kits for LDH, DAO and I-FABP demonstrated the expression of LDH, DAO, and I-FABP stimulated by LPS was suppressed by RFT (P < 0.01) in a dose-dependent manner (Figure 2a-c). Decreased TUNEL-positive cells by RFT in LPS-induced IEC-6 cells along with increased level of Bcl2 and decreased levels of Bax, cleaved caspase3, and cleaved caspase 9 showed that the apoptosis of LPS-induced IEC-6 cells was alleviated by RFT (P < 0.05) (Figure 3a-c). These data together indicate that RFT alleviates LPS-induced IEC-6 cell inflammation, cytotoxicity, and apoptosis.

RFT activates the autophagy of LPS-induced IEC-6 cells

**Figure 4.**
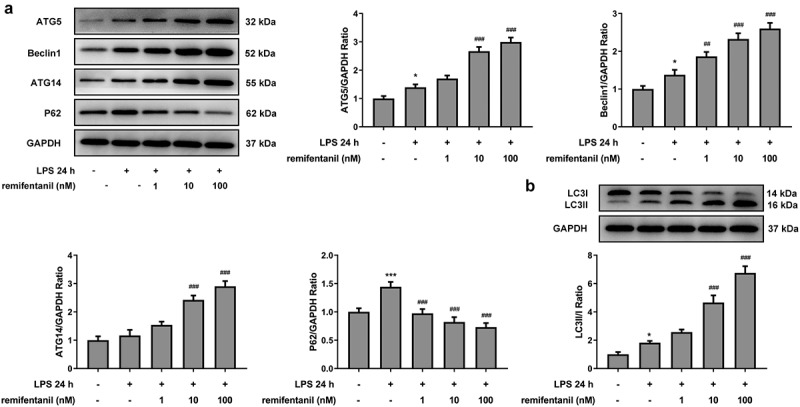
RFT activates the autophagy of LPS-induced IEC-6 cells. (a) The expression of autophagy-related proteins and (b) that of LC3II/LC3I was measured in LPS-induced IEC-6 cells. The experimental data are displayed as mean ± SD. *P < 0.05, ***P < 0.001 versus control. ##p < 0.01, ### p < 0.001 versus LPS

By analysis from in vitro/vivo experiments, several publications have consolidated their notion that the activation of autophagy can help protect IEC-6 cells from intestinal injury and that autophagy plays a protective role in multiple organ failure [27,28]. Therefore, we inferred that RFT may exert suppressive impacts on the inflammation and cytotoxicity by activating autophagy. The expression of autophagy-related proteins was then measured to observe whether RFT can activate autophagy of LPS-induced IEC-6 cells (Figure 4a). Interestingly, the levels of ATG5, beclin1, and ATG14 were increased by LPS (P < 0.05), which was reversed by increasing concentrations of RFT (P < 0.01) (Figure 4b). However, LPS stimulated the expression of P62, whereas this trend was abolished gradually after RFT treatment. We also found that the ratio of the relative expression of LC3 II and LC3I increased by LPS was further elevated by RFT. These results imply that RFT activates autophagy of LPS-induced IEC-6 cells.

Inhibiting autophagy blocks the ability of RFT to alleviate LPS-induced IEC-6 cell inflammation

**Figure 5.**
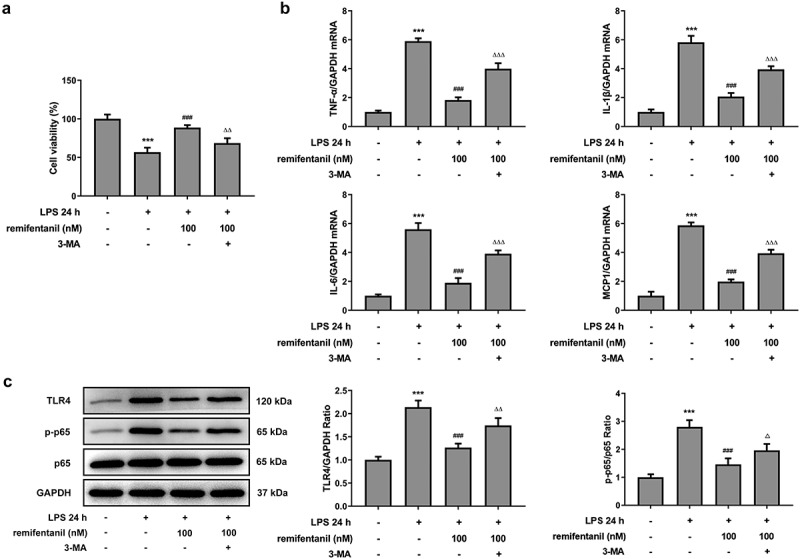
Inhibiting autophagy blocks the ability of RFT to alleviate LPS-induced IEC-6 cell inflammation. (a) The cell viability, (b) inflammatory factors and (c) inflammation-related protein expression was respectively measured by CCK-8, RT-qPCR and western blot in LPS-treated IEC-6 cells under RFT and 3-MA challenge. The experimental data are displayed as mean ± SD. ***P < 0.001 versus control. ### p < 0.001 versus LPS. ΔΔp<0.01, ^ΔΔΔ^p<0.001 versus LPS+ remifentanil

To confirm whether RFT exerts inhibitory effects on the cellular functions of LPS-induced IEC-6 cells via activating autophagy, 3 mM 3-MA was added in LPS-induced IEC-6 cells treated with RFT. As shown in Figure 5a, the cell viability enhanced by RFT (P < 0.001) in LPS-induced IEC-6 cells was abrogated by 3-MA (P < 0.01). Similarly, the contents of TNF-&alpha;, IL-1&beta;, IL-6, and MCP1 suppressed by RFT (P < 0.001) were released after 3-MA was used on LPS-induced IEC-6 cells (Figure 5b). The levels of inflammation-related factors suppressed by RFT in LPS-induced IEC-6 cells were enhanced by 3-MA (P < 0.05) (Figure 5c). Overall, these results indicate that inhibiting autophagy blocks the ability of RFT to alleviate LPS-induced IEC-6 cell inflammation.

Inhibiting autophagy blocks the ability of RFT to alleviate LPS-induced IEC-6 cell cytotoxicity and apoptosis

**Figure 6.**
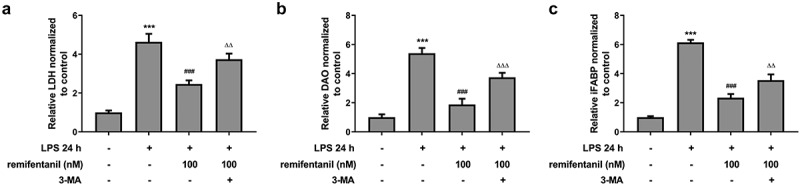
Inhibiting autophagy blocks the ability of RFT to alleviate LPS-induced IEC-6 cell cytotoxicity. (a) The levels of LDH, (b) DAO and (c) I-FABP were measured to indicate the changes of intestinal cytotoxicity. The experimental data are displayed as mean ± SD. ***P < 0.001 versus control. ^###^ p < 0.001 versus LPS. ^ΔΔ^p<0.01, ^ΔΔΔ^p<0.001 versus LPS+ remifentanil

**Figure 7.**
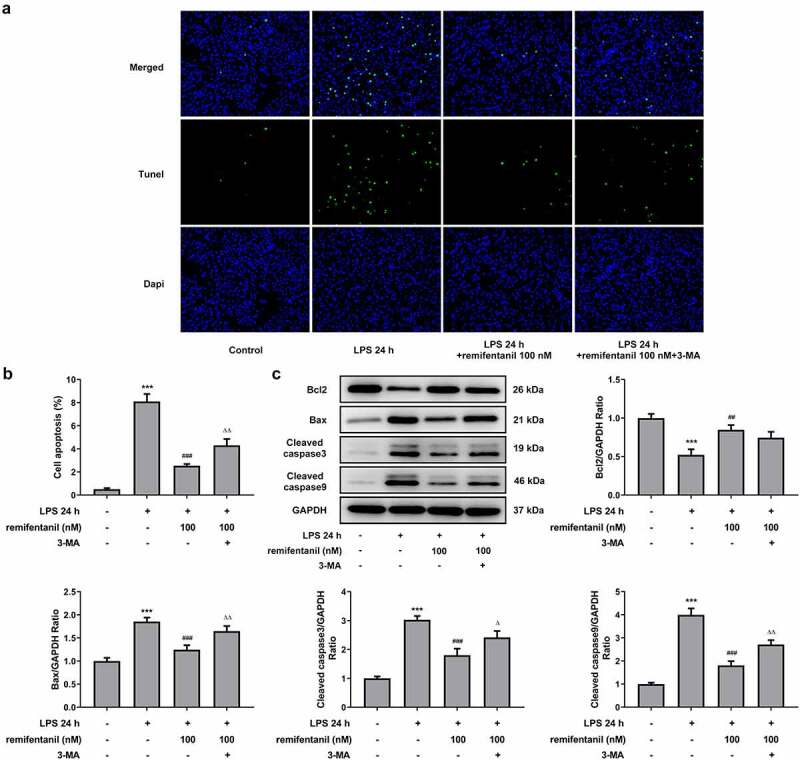
Inhibiting autophagy blocks the ability of RFT to alleviate LPS-induced IEC-6 cell apoptosis. (a) The expression of autophagy-related proteins and (b) that of LC3II/LC3I was measured in LPS-induced IEC-6 cells. The experimental data are indicated as mean ± SD. ***P < 0.001 versus control. ^##^ p < 0.01, ^###^ p < 0.001 versus LPS. ^Δ^p<0.05, ^ΔΔ^p<0.01 versus LPS+ remifentanil

Then, we detected whether cell cytotoxicity and apoptosis were influenced by RFT in LPS-induced IEC-6 cells via activating autophagy. Figure 6a-c displayed that the restorative effects of RFT on the increased LDH release and intestinal injury indicators including DAO and I-FABP of LPS-induced IEC-6 cells were abolished by 3-MA (P < 0.01). In addition, the apoptosis of IEC-6 cells markedly enhanced by LPS was reduced by RFT (P < 0.01), which was abrogated by further treatment of 3-MA (Figure 7a). Similarly, RFT brought about enhanced expression of Bcl2 and decreased expression of Bax, cleaved caspase3, and cleaved caspase 9, which was reversed when 3-MA was further added (P < 0.05) (Figure 7b, c). Taken together, inhibiting autophagy blocks the ability of RFT to alleviate LPS-induced IEC-6 cell cytotoxicity and apoptosis.

### Discussion

RFT is a p &omicron; tent μ-opioid analgesic with advantages in rapid blood–brain barrier equilibration time over other opioids [29]. Currently, besides the commonly known acute opioid tolerance and postoperative hyperalgesia that RFT can achieve, people usually make a connection between the use of RFT and rapid deep sedation and pain relief for patients with various injuries [30,31]. Some scholars, albeit with no direct evidence provided by experiments, have taken it plausible that RFT can reduce the risk of acute kidney injury [32]. Evidence has also suggested that RFT can reduce the apoptosis of myocardial cells to prevent the myocardial ischemia-reperfusion injury through Fas apoptosis signaling pathway [33]. In the present study, we detected the cell viability of LPS-treated IEC-6 cells challenged by RFT, and found that RFT exerted favorable impacts on the cell viability of these cells.

Studies in the past decade have pointed to the importance of autophagy in inflammatory and autoimmune diseases. The present work found that RFT was able to enhance autophagy to ameliorate LPS-induced IEC-6 cells injury evaluated by the detection of inflammatory indicators, intestinal injury indicators, and apoptosis levels. Autophagy initiates by the consecutive nucleation and elongation of double-membraned autophagosomes, which wraps partial of the cytoplasm in the course [34]. Afterward, autolysosomes are formed by the fusion of autophagosomes with lysosomes, during which LC3-II is also degraded by lysosomal proteases [35]. Then, sequestered intra-autophagosomal components will undergo the process of degradation by lysosomal hydrolases [35]. During this process, the autophagy state can be judged by p62 protein and LC3B-I/II. In the present study, the increased level of p62 together with transformation from LC3B-I to LC3B-II was noticed, indicating that autophagy was activated by RFT treatment in LPS-treated IEC-6 cells. The regulation of RFT for autophagy has been found in some studies [15,36,37]. A study found that RFT ameliorated hypoxia–reoxygenation-induced apoptosis via autophagy activation [38]. There was also a study reporting that LPS-induced cell death could be prevented by RFT [36]. Then, we investigated whether the cell viability, inflammation, and apoptosis of LPS-treated IEC-6 cells were influenced by RFT via cell autophagy. Intriguingly, 3-MA played reversal effects on the amelioration of RFT in the cell viability, inflammation, and apoptosis of LPS-treated IEC-6 cells.

### Conclusion

To conclude, this paper is the first to present evidence that RFT attenuates LPS-induced inflammation, the levels of intestinal injury indicators and apoptosis levels in IEC-6 cells by inducing autophagy in vitro, which will provide instructions for the future investigations into the use of RFT in the treatment of intestinal injury.

## Highlights

1. Remifentanil prevents IEC-6 cells from LPS-induced cytotoxicity and injury.

2. Remifentanil reduces inflammation and apoptosis caused by LPS.

3. Remifentanil enhanced autophagy to ameliorate intestinal injury.
